# Structure of the BTB Domain of Keap1 and Its Interaction with the Triterpenoid Antagonist CDDO

**DOI:** 10.1371/journal.pone.0098896

**Published:** 2014-06-04

**Authors:** Anne Cleasby, Jeff Yon, Philip J. Day, Caroline Richardson, Ian J. Tickle, Pamela A. Williams, James F. Callahan, Robin Carr, Nestor Concha, Jeffrey K. Kerns, Hongwei Qi, Thomas Sweitzer, Paris Ward, Thomas G. Davies

**Affiliations:** 1 Astex Pharmaceuticals, Cambridge, United Kingdom; 2 GlaxoSmithKline, King of Prussia, Pennsylvania, United States of America; 3 GlaxoSmithKline, Stevenage, United Kingdom; 4 GlaxoSmithKline, Collegeville, Pennsylvania, United States of America; Van Andel Research Institute, United States of America

## Abstract

The protein Keap1 is central to the regulation of the Nrf2-mediated cytoprotective response, and is increasingly recognized as an important target for therapeutic intervention in a range of diseases involving excessive oxidative stress and inflammation. The BTB domain of Keap1 plays key roles in sensing environmental electrophiles and in mediating interactions with the Cul3/Rbx1 E3 ubiquitin ligase system, and is believed to be the target for several small molecule covalent activators of the Nrf2 pathway. However, despite structural information being available for several BTB domains from related proteins, there have been no reported crystal structures of Keap1 BTB, and this has precluded a detailed understanding of its mechanism of action and interaction with antagonists. We report here the first structure of the BTB domain of Keap1, which is thought to contain the key cysteine residue responsible for interaction with electrophiles, as well as structures of the covalent complex with the antagonist CDDO/bardoxolone, and of the constitutively inactive C151W BTB mutant. In addition to providing the first structural confirmation of antagonist binding to Keap1 BTB, we also present biochemical evidence that adduction of Cys 151 by CDDO is capable of inhibiting the binding of Cul3 to Keap1, and discuss how this class of compound might exert Nrf2 activation through disruption of the BTB-Cul3 interface.

## Introduction

Keap1 (Kelch-like ECH-associated protein 1) is a multi-domain protein which plays a key role in the regulation of Nrf2, a transcription factor that mediates the expression of a large array of cytoprotective enzymes in response to electrophilic and oxidative assault [1]–[4]. In common with related family members, it acts in concert with members of the CRL3 class of Cullin-RING-Ligase E3 ligases to provide substrate-specific recruitment for ubiquitination, and consists of a three domain architecture composed of an N-terminal BTB (Broad complex, Tramtrack, and Bric-a-Brac) domain, an intervening region (IVR) or BACK domain, and a C-terminal Kelch repeat domain [1], [5], [6]. Although X-ray crystallographic information for Keap1 has been limited to its Kelch domain, structures for two related proteins, namely KLHL3 [7] and KLHL11 [8], have provided confirmation that the BTB and BACK domains together provide a binding platform which engages the N-terminal domain of the E3 ubiquitin ligase Cul3/Rbx1 and act as an adaptor between substrate recognition and the ubiquitination machinery [9]. C-terminal to the IVR, the β-propeller Kelch domain is a protein-protein interaction module which recognises and interacts with motifs on the Nrf2 substrate [10], [11]. Keap1 is known to dimerize through its BTB domain [12], and models of the mechanism of action require dimerization for constructive engagement with the Nrf2 substrate [13]. This dimerization has also been observed crystallographically for structures of the other BTB domains solved to date [5], [14].

In the case of Keap1, the BTB domain is unique in providing an additional role in the sensing of oxidative stress [1], [15]. The human body is continuously exposed to a range of electrophilic and oxidative species which can cause damage to cellular components such as lipids, proteins and nucleic acids. Such oxidative damage can lead to chronic inflammation, tissue degeneration and loss of function, and cells have a requirement to respond dynamically to these threats in order to minimize their detrimental effects.

The Keap1/Nrf2 system has evolved as one such response mechanism, allowing the upregulation of various cytoprotective proteins in order to exert an antioxidant effect when required. Under basal conditions, Keap1 acts to negatively regulate Nrf2, sequestering it through interaction via the Kelch domain and leading to its ubiquitination (and subsequent proteasomal degradation) as a consequence of its resulting proximity to Cul3/Rbx1. Increased levels of oxidative or electrophilic stress have been shown to result in covalent modification of key cysteine residues in the BTB and BACK domains [3], [15]–[21] leading to dissociation of Cul3, and potentially other conformational changes that cause loss of productive Nrf2 binding [1], [22], [23]. As a result of these changes, Keap1 mediated ubiquitination of Nrf2 is perturbed and levels of free Nrf2 rise. Nrf2 can then translocate to the nucleus where it dimerizes with a small Maf protein and acts upon the antioxidant response element (ARE) in the regulatory region of its target genes. The result is an increased expression of proteins that have a protective effect for the cell such as NAD(P)H:quinone oxidoreductase 1, glutathione-S-transferase and heme-oxygenase-1 [24], [25].

This ability of Keap1/Nrf2 to respond to oxidative stress affords protection against excessive damage and inflammation which could be detrimental for normal cellular function [6]. There is evidence that there are genetic determinants of sensitivity and disease-causing potential of increased levels of oxidative stress, and *Nrf2^−/−^* mice have been shown to be more susceptible to inflammation in response to cigarette smoke [26]–[29]. In certain disease pathologies additional stimulation of the pathway may be beneficial, and Keap1 is increasingly being recognized as a potential target for therapeutic intervention in the treatment of a range of diseases involving oxidative stress and inflammation [30], [30]–[35]. A number of small molecule antagonists of Keap1 are known, the majority of which are electrophiles believed to function by covalent modification of the Keap1 cysteine residues responsible for sensing oxidative stress [35]–[39].

Derivatives of the triterpenoid compound 2-cyano-3,12-dioxooleana-1,9-dien-28-oic-acid (CDDO) form a well-studied group of anti-inflammatory compounds which exert their effects through inhibition of Keap1 [40]. For example, the methyl ester CDDO-Me (bardoxolone-methyl; Reata/Abbott) was until recently in phase III clinical trials for diabetics with chronic kidney disease, whilst the imidazole amide analogue CDDO-Im has been shown to reduce cigarette smoke-induced emphysema in an Nrf2-dependent fashion and is active in animal models of chronic obstructive pulmonary disease (COPD) [26]. Other Keap1 antagonists include the naturally occurring isothiocyanate sulforaphane [41], and the compound Tecifidera (BG-12/dimethyl fumarate; Biogen Idec) which has recently been approved by the FDA for the treatment of multiple sclerosis, and believed to operate (at least in part) via Keap1-mediated activation of the Nrf2 pathway [42].

Two main hypotheses have currently been proposed to explain the ability of Keap1 cysteine covalent modification (as a result of oxidative stress, or by therapeutic agents) to activate the Nrf2 pathway. In the first model (“hinge and latch”), the modification of one or more cysteines in the BTB and/or BACK domains (most notably Cys 151, Cys 273, Cys 288) leads to the partial disruption of the Nrf2/Kelch interaction, thus preventing the correct engagement of the Nrf2 substrate and Cul3/Rbx1. Nrf2 remains anchored to a single Kelch domain of the Keap1 dimer through its ETGE motif, but nascent synthesis leads to Nrf2 accumulation and subsequent activation of genes under the control of the ARE [13], [43].

An alternative hypothesis is based on the observation that modification of the highly reactive Cys 151 decreases the binding of Cul3 to Keap1, leading to loss of Nrf2 ubiquitination [22], [23]. Mass spectrometry studies have shown that Cys 151 is the only Keap1 cysteine residue to be consistently and highly modified by Nrf2 activators [36], [44], and its predicted proximity to the Cul3 binding interface is consistent with this Cul3-dissociation model [7]. However, a recent study in live cells has questioned whether any dissociation of Cul3 from Keap1 is physiologically relevant [45], and there are conflicting conclusions as to the relative importance of modification at the Cys 151 position in pathway activation versus other cysteine residues [23], [46], [47].

A full characterization and understanding of the mode of action and design of these compounds has been hampered by a lack of structural information on their possible interaction with Keap1. Although a low resolution single particle electron microscopy structure has been reported for full-length Keap1 [48], there is currently no crystal structure for the Keap1 BTB domain. Structural information is available for the BTB domains from other related proteins [5], but Keap1 is the only family member which contains a cysteine at position 151, a reflection on its specific role as a dynamic sensor of electrophilic stress in its environment [49].

We report here the structure of the Keap1 BTB domain, both in the apo state, and complexed with CDDO/bardoxolone, which is the more soluble carboxylic acid form of the widely studied Keap1 antagonist CDDO-Me/bardoxolone-methyl. As well as confirming the overall fold of the protein, these allow structural confirmation of the interaction of CDDO with the BTB domain, and an unambiguous understanding of its interactions with the Cys 151 binding site. As well as providing important information for the structure-based design of modulators of Keap1 function, the structure also provides a basis for understanding how this class of compounds might exert their activatory effects through disruption of the Keap1-Cul3 interface. In addition, we also report here the structure of a mutant form of the Keap1 BTB domain where the reactive cysteine at position 151 has been mutated to tryptophan. This mutation has been shown to constitutively activate the Nrf2 pathway through antagonism of Keap1, and we discuss this structure in the context of both apo and CDDO-bound forms of the protein.

## Results

Initial attempts to obtain diffraction-quality crystals of the isolated BTB domain (residues 48–180) of Keap1 were unsuccessful, and this was hypothesised to be due to conformational flexibility and heterogeneity, particularly in the C-terminal region (residues 165–180) of the BTB domain which is likely to be stabilized by packing with helices of the BACK domain in full-length Keap1. In order to identify a construct more amenable to crystallization, a number of point mutations were made to the C-terminal region of BTB, and the effect on the thermal stability of these mutant proteins was studied using a fluorescence-based thermal denaturation assay. Of these, S172A, which exhibited a ΔT_m_ of >3°C relative to the wild-type (Figure S1 in File S1), was observed to give rise to crystals which diffracted to ∼2.4 Å resolution and allowed its subsequent structure determination. The position of this conservative mutation is >15 Å from the key Cys 151, and is unlikely to affect the structural interpretation. In addition, the presence of the S172A mutation in a longer construct (residues 35–318), which included the BACK domain, was found to have no effect on its ability to bind to Cul3 in a direct binding assay, suggesting that the overall structure has not been significantly perturbed (Figure S2 in File S1).

**Figure 1:**
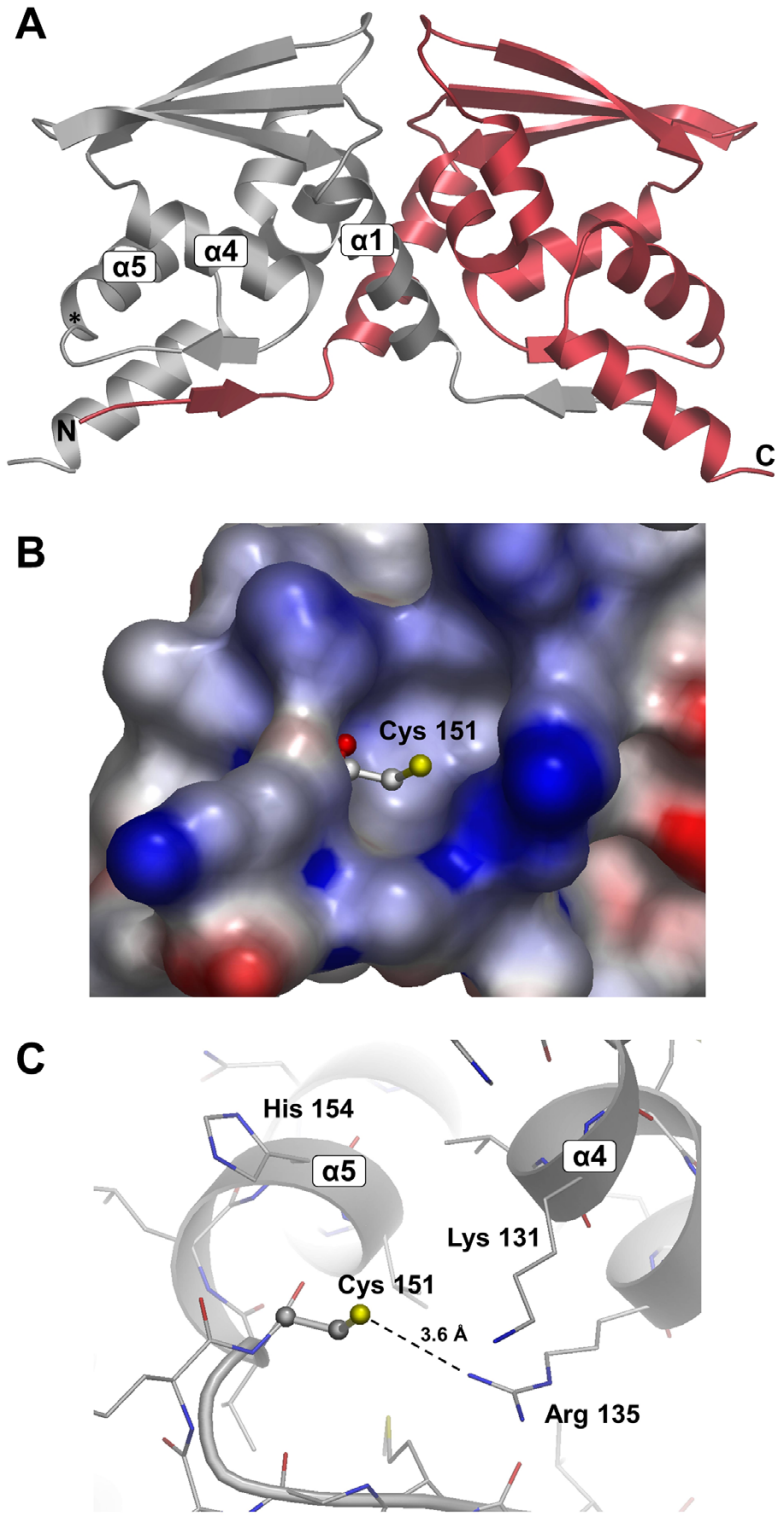
The structure of Keap1 BTB. (A) Overall fold of the Keap1 BTB crystallographic dimer as a cartoon representation. The N and C-termini, and key alpha-helical secondary structural elements are labelled for one BTB monomer. The approximate position of Cys 151 is marked with an asterisk. (B) Surface around Cys 151 coloured according to electrostatic potential. Blue regions indicate areas of positive potential and red regions areas of negative potential as calculated by AstexViewer [70]. (C) Details of Cys 151 environment showing close contact with Arg 135. Note that the side-chains of Lys 131 and and Lys 150 are highly flexible and exhibit very weak electron density. Some disorder is also evident for Arg 135.

In common with previously described structures from both the BTB-ZF and BTB-BACK-Kelch sub-families, the structure of Keap1 BTB crystallizes as a dimer, with the two domains related by a crystallographic two-fold axis (Figure 1A). The overall structure resembles that of other BTB domains, and consists of a three-stranded β-sheet flanked by six α-helices, the first of which (α1) forms a key part of the dimerization interface. In common with many (but not all) other BTB domains, the far N-terminal residues form a domain swapped β-sheet with residues 143–149 of the symmetry partner and also contribute to the dimerization interface of the two molecules. Cys 151 is located at the tip of a flexible loop adjacent to helix 5 (α5) in a solvent exposed depression, and surrounded by a cluster of positively charged residues (His 129, Lys 131, Arg 135, Lys 150, His 154), although the orientation of the lysine side-chains is unclear due to flexibility.

As suggested previously based on homology modelling [49], it is possible that these residues play a role in activating Cys 151 by lowering its pK_a_ and stabilizing the thiolate anion. The immediate environment of the cysteine exhibits a positive electrostatic potential (Figure 1B), and although the electron density suggests a large degree of mobility, the side-chain of Arg 135 appears to be predominantly orientated towards Cys 151 (Figure 1C) with which it forms a relatively close contact (r_Nη1…Sγ_∼3.6 Å). In general, the loop containing Cys 151 exhibits higher B-factors and weaker electron density than the core of the protein, and we speculate that this flexibility may be relevant to the role of this region in driving the transition between apo and Cul3 bound states. A high degree of flexibility is also observed for residues 114–118, which contains the Φ-x-E motif, a key region for interaction with Cul3 [7], [50]. Residues in this loop have been observed to be disordered in several uncomplexed BTB structures including Gigaxonin [51] and KLHL11 [8], but have been shown to become ordered upon interaction with Cul3.

### Structure of Keap1 BTB domain in complex with CDDO

**Figure 2:**
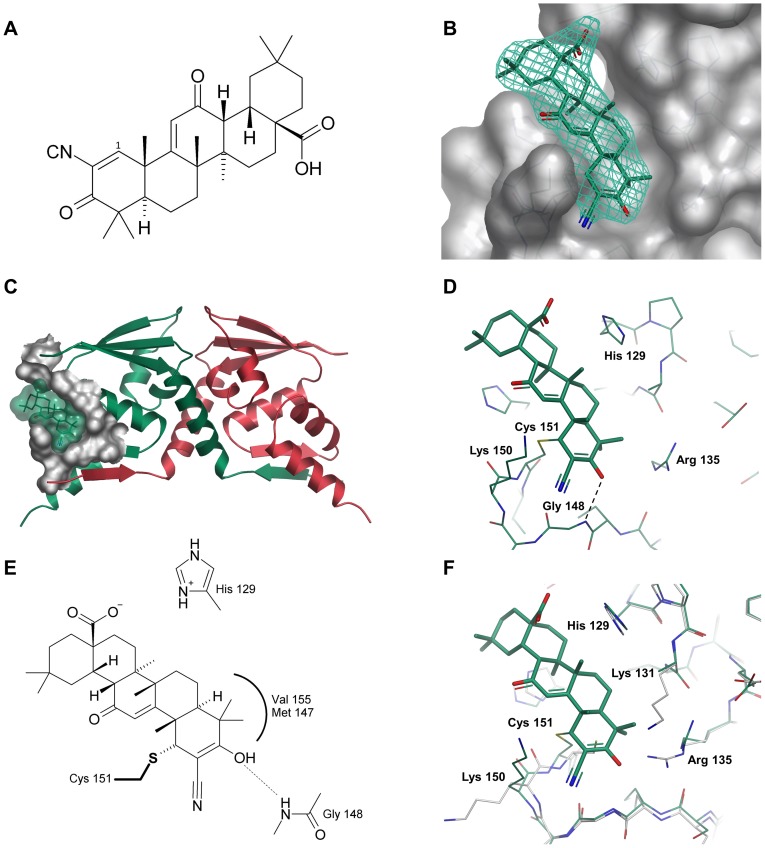
CDDO and its interaction with the Keap1 domain of BTB. (A) Chemical structure of CDDO/bardoxolone. (B) Surface representation of Keap1 BTB with CDDO bound in region of Cys 151. The final 2mF_o_-DF_c_ electron density (contoured at 1σ) is shown as a green mesh. (C) Overview of CDDO binding in context of the BTB crystallographic dimer (only one binding site is shown for clarity). The surface of the CDDO binding site is shown in grey. (D) Details of covalent and non-covalent interactions between CDDO and BTB. Hydrogen bonds are denoted by dashed lines. The side-chain of Lys 131 is not visible in the electron density and has been truncated to the Cβ atom. (E) Schematic diagram of interactions between CDDO and BTB. His 129 is shown in the protonated state to emphasize its potential to form an electrostatic interaction with the carboxylate of CDDO. (F) Overlay of apo (white carbons) and CDDO-bound BTB (green carbons) in region of Cys 151.

The Keap1 antagonist CDDO-Me is poorly soluble in aqueous solution, and so we chose to study the binding of the corresponding carboxylic acid CDDO which is more soluble (Figure 2A). After soaking with CDDO there was clear evidence for covalent modification of Cys 151, with electron density supportive of ordered compound binding. The antagonist occupies a shallow groove containing Cys 151 (Figures 2B and 2C), which has become deeper and more defined upon compound binding. As anticipated, the Sγ of Cys 151 is observed to form a covalent bond with CDDO by Michael addition to the electrophilic *sp^2^* carbon β to the cyano group (Figure 2D), creating a new chiral centre at position 1 with (*R*) stereochemistry. It should be noted that although the bound CDDO has been built as the enol tautomer, the shape of the electron density at this resolution (particularly for the cyano functionality, which is only weakly defined) precludes a definitive assignment over the keto form. No modification of other cysteine residues present in the crystallographic construct (ie Cys 77 and Cys 171) is apparent from the electron density, in agreement with the observation that Cys 151 possesses increased reactivity.

Apart from this covalent bond, there are few specific polar interactions with the protein, and the B-factors for the ligand and surrounding residues are high relative to other protein regions, suggesting a large degree of mobility. A single, hydrogen bond is formed between the backbone amide nitrogen of Gly 148 and the enolic oxygen (r_O…N_ = 3.3 Å), whilst the carboxylic acid occupies a region close to the side-chain of His 129 with which it could potentially form a weak electrostatic interaction. The remainder of the interactions with BTB are hydrophobic and steric, with the ring systems accommodated in a groove formed by the side-chains of His 154, His 129, Tyr 85, and the C-4 *gem*-dimethyl group occupying a small cavity near the side-chain of Val 155 and the hydrophobic portion of Lys 131. Interactions formed between CDDO and BTB are summarized schematically in Figure 2E. Based on the relatively solvent exposed position of the carboxylate group of CDDO, any differences in binding between the acid form of CDDO and other derivatives (eg CDDO-Me, CDDO-Im) are expected to be relatively minor.

A comparison of the apo form of BTB with that bound to CDDO shows a number of changes associated with compound binding (Figure 2F). The side-chain of Arg 135 changes rotamer to avoid a clash with the C-4 *gem*-dimethyl group, and the side-chain of Lys 131, which is already poorly defined in the apo structure, is no longer visible in the electron density for the CDDO-bound form, presumably because it becomes disordered upon displacement from its original position. There are also more subtle changes in the immediate region of Cys 151 including a ∼2.8 Å outward movement of the cysteine's Sγ atom upon formation of the covalent bond with CDDO, and an associated displacement by ∼1.5 Å of the surrounding peptide backbone.

**Figure 3:**
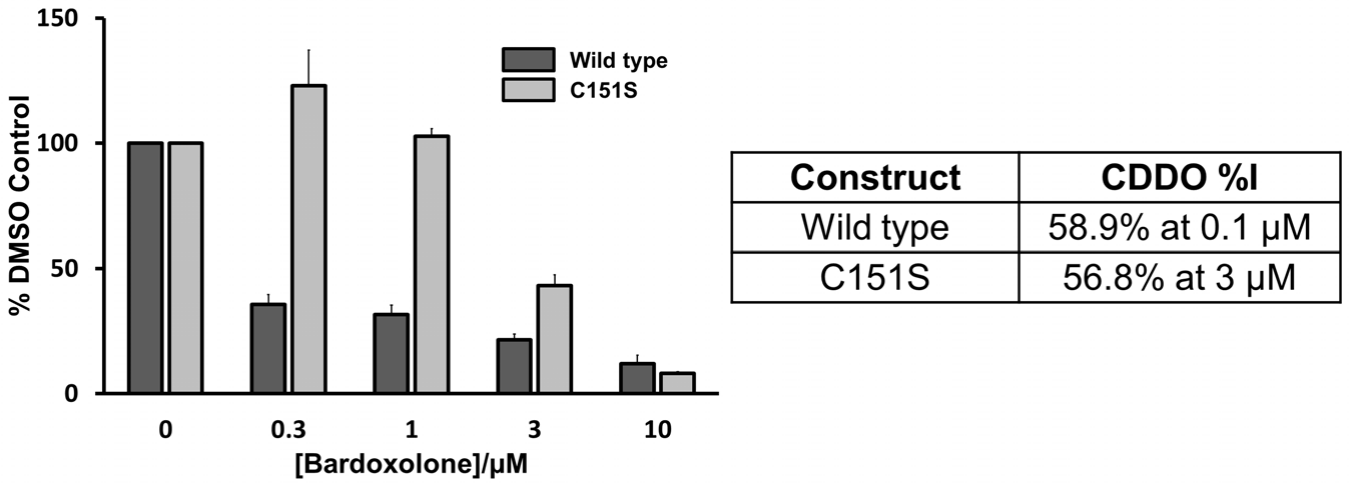
Inhibitory effects of CDDO on Cul3 binding to wild-type C151 and C151S BTB-BACK as measured by AlphaScreen proximity assay. The change in signal as a function of CDDO concentration is reported relative to a DMSO control, and is the mean of three measurements. Error bars represent the standard deviation.

The ability of other covalent modifiers (eg tBHQ, sulforaphane, IAB) to disrupt the BTB/Cul3 interaction has been reported previously [4], [22], [52], [53]. In order to confirm that CDDO has the potential to operate by a similar mechanism, we examined its ability to inhibit the binding of Cul3 to BTB using an AlphaScreen bead-based proximity assay (PerkinElmer). We chose a minimal Keap1 construct capable of Cul3 binding for this study (BTB with only the first ∼50 residues of the BACK domain (residues 35–235) and designed to exclude Cys 273 and Cys 288), in order to establish whether covalent adduction to Cys 151 alone had the ability to inhibit the interaction of the protein partners. A clear dose-dependent inhibition of Cul3 binding to Keap1 by CDDO was observed, with an IC_50_<100 nM (Figure 3). In order to demonstrate the importance of Cys 151 as the primary point of interaction with this construct, the study was repeated using a C151S mutant form of Keap1, which retains its ability to bind Cul3 [1], but was expected to be incapable of binding CDDO. Although there remains an apparent inhibitory effect at higher concentrations (>1 µM), the results clearly show a significant difference in behaviour between the wildtype and C151S constructs, and are consistent with adduction of Cys 151 being the primary driver for inhibition of Cul3 binding.

**Figure 4:**
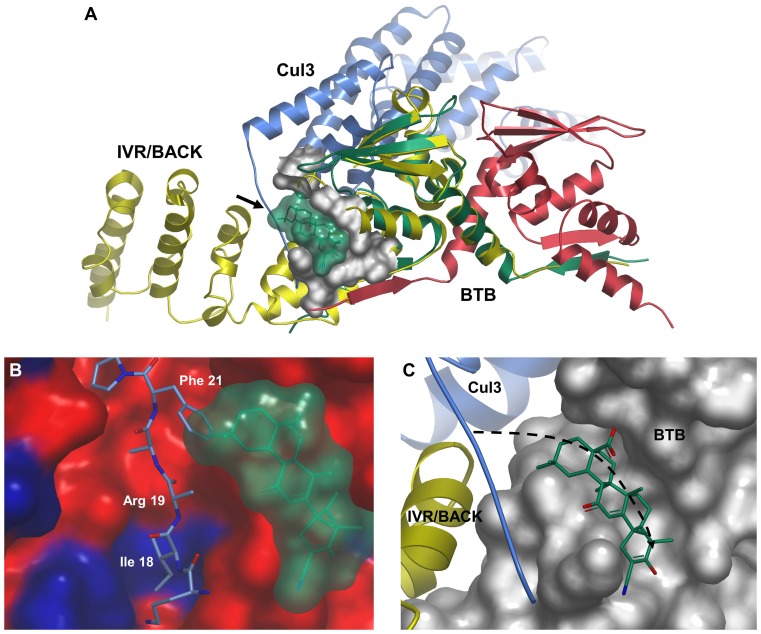
Position of Keap1-bound CDDO in the context of possible Cul3 binding surfaces. (A) Superposition of KLHL11/Cul3 (PDB code 4ap2) and BTB-CDDO showing proximity to Cul3 N-terminal tail (marked with arrow). KLHL11 is shown in a yellow cartoon representation, Cul3 in blue and the Keap1 BTB dimer in red and green. The CDDO binding site is highlighted as a grey surface, with the surface of CDDO shown in green. For clarity, bound CDDO is only shown for one BTB monomer, and a single copy of KLHL11/Cul3 is shown, although the KLHL11/Cul3 complex dimerizes through the KLHL11 BTB domain in the crystal lattice. (B) Surface representation of the KLHL11 Cul3 binding groove coloured by amino acid sequence identity with Keap1 (blue  =  identical, red  =  non-identical). Keap1 and KLHL11 sequences were aligned using ClustalW [71] to determine regions of identity. The Cul3 N-terminal strand is depicted with blue carbons, and CDDO is shown as a surface representation in green. The position of CDDO was taken from that of its complex with BTB when overlaid with the KLHL11/Cul3 structure. The side-chain of Arg 19 is shown truncated to Cβ as deposited with the PDB. (C) Surface representation of BTB in the region of the CDDO binding groove, showing potential alternative path for Cul3 N-terminal tail upon binding to Keap1 (dashed arrow). The Cul3 and the IVR/BACK regions are taken from the superposition of the KLHL11/Cul3 structure with Keap1 BTB.

In order to rationalise how the binding of CDDO to Cys 151 might exert its effect on the binding of Cul3, we firstly overlaid the BTB-CDDO structure with the previously solved structure of KLHL11 (which includes both BTB and BACK domains) complexed with Cul3 [8]. The overlay positions Cys 151 proximal to a hydrophobic groove in the “3-box” motif of the KLHL11 BACK domain which was identified as a key point of interaction with the N-terminal tail of Cul3 (Figure 4A), and important for KLHL11/Cul3 association [8]. An analysis of sequence conservation between the BACK domains of KLHL11 and Keap1 shows that key hydrophobic residues responsible for engaging Ile 18 of the Cul3 N-terminal tail in KLHL11 are conserved in Keap1 (Figure 4B). As this hydrophobic sub-pocket is likely to be an important energetic hotspot for the association of Cul3 with KLHL11, it is possible that full-length Keap1 interacts with Cul3 in a similar manner. Given the potential for CDDO to occupy a position adjacent to the Cul3 tail in Keap1, its binding to the Cys 151 region might have the potential to weaken or abrogate the binding of Cul3, either through direct steric hindrance with N-terminal Cul3 residues (*eg* Arg 19 or Phe 21), or indirectly through the conformational changes mentioned above. The precise extent to which CDDO would spatially impact upon Cul3 N-terminal residues is difficult to predict in the absence of structural information on the full Cul3/Keap1 complex, and could be more extensive than apparent from the superposition presented here.

Alternatively, we speculate that the N-terminal tail of Cul3 could follow a somewhat different path when bound to Keap1 compared to KLHL11, perhaps directly occupying the groove surrounding Cys 151 which is apparent in the CDDO-bound form (Figure 4C). This model has the advantage that modification of Cys 151 would more obviously lead to direct abrogation of binding of Cul3, particularly for smaller antagonists such as dimethyl fumarate (or simple oxidation) which would not be expected to directly clash with Cul3 residues if the N-terminus was bound as in the KLHL11 structure.

### Structure of Keap1 C151W mutant BTB

In order to gain further insight into mechanism of action of modifications to Cys 151, we also solved the structure of the Keap1 BTB domain C151W mutant. In the context of full-length Keap1, this mutation leads to significantly decreased interaction with Cul3 in pull-down experiments with cell-extracts [23], and is constitutively activating of Nrf2 *in vivo* [38]. In addition, we have recently demonstrated that introduction of the C151W mutation disrupts the interaction of Cul3 with full-length Keap1 using a TR-FRET assay (manuscript in preparation).

**Figure 5:**
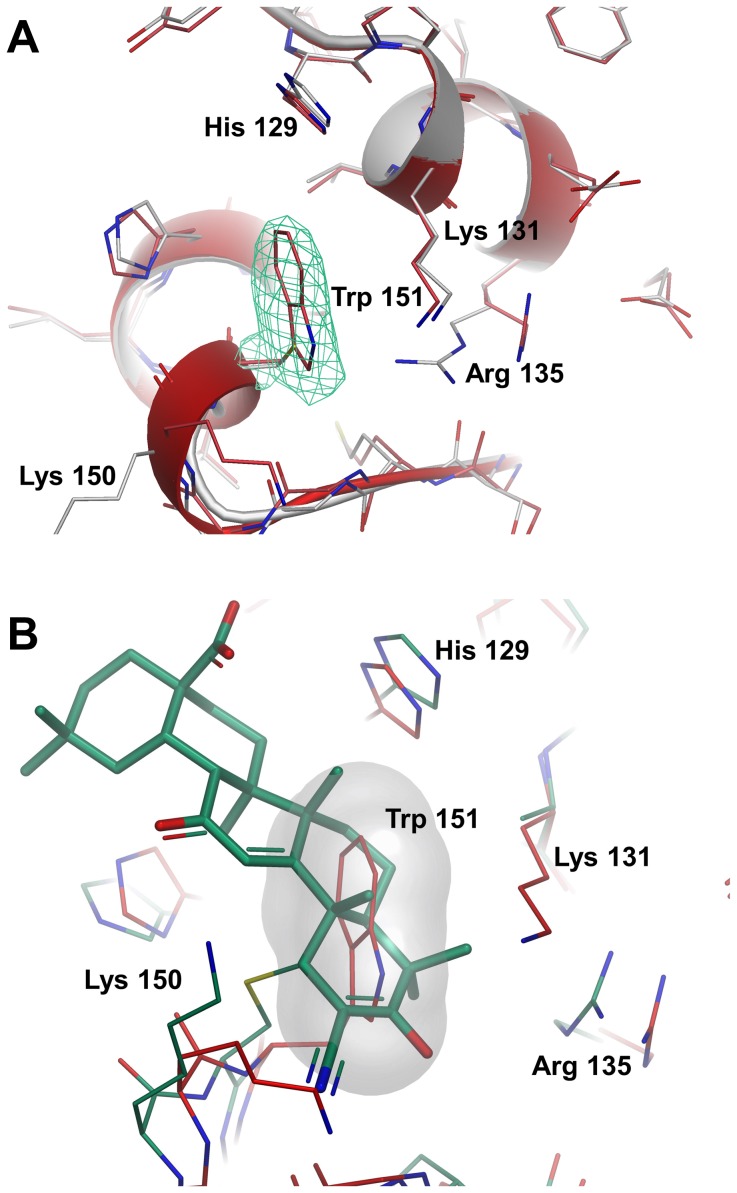
Comparison of Keap1 BTB C151W mutant with apo and CDDO-bound structures. (A) Overlay of Keap1 BTB C151 (white carbons) and C151W mutant (red carbons) in Cys 151 region. The final 2mF_o_-DF_c_ electron density (contoured at 1σ) is shown as a green mesh for the Trp 151 side-chain. (B) Overlay of the C151W BTB mutant (red carbons) and BTB-CDDO (green carbon) showing overlap between the antagonist and indole ring system of tryptophan. The volume occupied by the side-chain of Trp 151 has been highlighted as a white surface.

An overlay with the apo C151 structure reveals a change in rotamer for the side-chain of Arg 135, but no significant conformational movements with the potential to disrupt the binding with Cul3 (Figure 5A). In addition, the structure of the C151W mutant presented here shows no evidence for disruption of the BTB homodimerization interface. Superposition of the C151W and CDDO-bound structures shows that the side-chain of Trp 151 adopts a rotamer in which the indole ring is orthogonal to the CDDO ring system, although occupying a similar region of space to the lower portion of the antagonist (Figure 5B). Unlike CDDO, the tryptophan side-chain is not positioned such that it would be expected to clash directly with the N-terminal tail of Cul3 in the KLHL11-bound conformation, and although it is possible that the crystal structures presented here do not reveal the full conformational changes associated with this mutation in solution, and their potential to impact on Cul3 binding, the structure of C151W is more supportive of a model where the Cul3 N-terminal tail occupies the CDDO binding groove directly before mutation or covalent adduction of Cys 151. Further experimental evidence is clearly required to distinguish between these and other possibilities, and a more complete understanding of the effects of Cys 151 perturbation might benefit from structural information for a construct containing at least the first ∼50 residues of the BACK domain (the “3-box” motif), which are likely to be important for the Keap1-Cul3 interaction [8] and expected to be adjacent to Cys 151. Nevertheless, the structures presented here demonstrate that modifications to Cys 151 occur in close proximity to key surfaces which are likely to be responsible for tight association of the protein partners.

## Discussion

The structures described here provide the first atomic-level view of the BTB domain of Keap1, and the unique environment of Cys 151 which is the key residue responsible for detecting increased levels of oxidative stress. Keap1 is now recognized as a key drug target, and these structures provide the basis for the design of novel covalent as well as non-covalent antagonists of Keap1. The mechanism by which different classes of covalent modifiers of Keap1 exert their activatory effects on Nrf2 has been unclear, but we show here that CDDO is capable of covalent interaction with Cys151 and adopts a defined binding mode with the BTB domain.

In contrast to the C151W mutant described above, mutation to serine at this position is known not to inhibit the binding of Cul3 [23], and the ability of covalent modifications to elicit an inhibitory effect was observed to be correlated with their partial molar volume, possibly through a steric or conformational effect. Neither the binding of CDDO, nor the C151W mutation appears to lead to large conformational changes, and although we cannot preclude the possibility that additional effects are relevant in solution, or in full-length Keap1, the structures presented here suggest a model where dissociation of Cul3 may be driven by direct steric hindrance with residues in its N-terminal tail. Although there is still ambiguity over the precise binding surface responsible for engaging these Cul3 residues in its complex with Keap1, this study provides an insight into the molecular geometry adopted by Cys151 adducts in order to disrupt the interaction with Cul3. This empirical information could be used to help define more precisely the molecular envelope and binding epitope required for pharmacologically active covalent modifiers capable of activating the Nrf2 pathway.

In addition, the structural information provides a clear insight into complementary non-covalent interactions which might be engaged to increase both the affinity and selectivity of the covalent warhead, as well as providing a start-point for the design of purely non-covalent antagonists. The ability to obtain an apo crystal form for a therapeutic target is an important prerequisite for fragment-based approaches to drug discovery (FBDD) [54], and we recently highlighted the ability of X-ray crystallographic fragment screening to discover and exploit novel and unprecedented binding sites on proteins [55], [56]. The availability of the apo crystal structure of the BTB domain of Keap1 therefore offers the potential to assist in the identification of novel classes of antagonists against this important therapeutic target.

## Materials and Methods

### Expression and purification of Keap1

For the crystallographic work, cDNA encoding residues 48–180 of Human Keap1 (Uniprot Q14145) were subcloned into the vector pET28b to incorporate a thrombin-cleavable N-terminal hexahistidine tag. A single point mutation was introduced at residue 172 (S172A) as described above, and a second construct was made containing a C151W mutation in addition to S172A.

The protein was expressed in BL21 (DE3) cells, using 0.2 mM IPTG for overnight induction at 18°C. Harvested cells were resuspended in lysis buffer (50 mM Tris/HCl pH 8.0, 150 mM NaCl), and disrupted by sonication at 4°C before clarification by centrifugation. The crude protein was then purified by Ni-affinity chromatography (elution buffer 50 mM Tris/HCl pH 8.0, 150 mM NaCl, 250 mM imidazole), before overnight dialysis against 50 mM Tris/HCl pH 8.0, 150 mM NaCl at 4°C in the presence of bovine thrombin (Sigma; 1∶100 (w/w)). The protein was subsequently purified by size exclusion chromatography using a HiPrep S75 26/60 column (GE Healthcare) equilibrated in 25 mM Tris/HCl pH 8.0, 150 mM NaCl, and 1–3 mM TCEP, before concentration to 11 mg/mL and storage at −80°C.

For the AlphaScreen assay, slightly longer Keap1 constructs were used, which included part of the BACK domain (residues 35–235), for both the wild-type C151 and the C151S mutant. Protein expression and purification was carried out in a similar manner to that described above, but without cleavage of the N-terminal His-tag.

### Expression and purification of Cul3

Rbx1 protein was co-expressed with Cul3 to aid in stability of the Cul3 protein. Baculovirus constructs for FLAG-His-Tev-Avi tagged Cul3 (residues 1–768) and Rbx1 (1–108) were generated separately and then co-expressed in Sf9 cells. The cells were disrupted by pressure lysis in a buffer containing 50 mM Tris, 300 mM NaCl, 10% glycerol, 0.5 mM TCEP, pH 7.5 and centrifuged at 38000 *g* for 45 min at 4°C. The supernatant was bound in batch mode with Qiagen NiNTA SF beads (Valencia, CA) for 2 h at 4°C with gentle rotation before washing with 30 mM imidazole in lysis buffer and elution with 200 mM imidazole in lysis buffer. The eluted protein was desalted on Zeba desalting column (Thermo Scientific, Rockford, IL) into BirA biotinylation buffer (10 mM Tris, pH 8.5, 10 mM ATP, 10 mM Mg(OAc)_2_, 0.5 mM Biotin). TEV protease (1∶50 TEV∶protein by mass) and BirA (2.5 µg BirA/nmol Cul3) were added to the protein solution and incubated at 4°C overnight. Tag cleavage and protein biotinylation was confirmed by LC/MS the next day. The protein was further purified by size exclusion chromatography using a Superdex 200 column.

### Synthesis of CDDO

CDDO for the crystallographic work was prepared using literature procedures [57]–[60]. The CDDO used in the AlphaScreen assay was purchased from Cayman chemical.

### Crystallization and Data collection

Crystallization was carried out by hanging drop vapour diffusion ([BTB] = 11 mg/mL; 1∶1 BTB∶reservoir ratio), and crystals with narrow needle morphology were observed to grow from a wide range of PEG/salt conditions. The crystals used to determine the structures described here grew from 0.2 M lithium acetate and 18–21% PEG 3350 at 20°C, and apo crystals reached an average size of 200 µm×10 µm×5 µm in 3–7 days. The CDDO complex was generated by co-crystallization using the same conditions. CDDO was added to BTB to a nominal 3 mM final concentration, although there was some evidence for compound insolubility, and incubated on ice for approximately 2 hours prior to setting up crystallization drops. All crystals were cryoprotected by brief immersion in a solution containing the well buffer to which glycerol had been added to a final concentration of 15% (v/v), before plunge-freezing into liquid nitrogen. Data were collected for the apo crystal and the CDDO co-crystal on ID29 at the European Synchrotron Radiation Facility, and for the C151W mutant on I04-1 at the Diamond Light Source. Data collection statistics are presented in Table 1.

**Table 1:** X-ray data collection and refinement statistics.

	BTB apo	BTB-CDDO	BTB C151W
**Data collection**			
Beamline	ESRF ID29	ESRF ID29	DLS I04-1
Space group	*P*6_5_22	*P*6_5_22	*P*6_5_22
Cell dimensions			
*a*, *b*, *c* (Å)	42.81, 42.81, 266.51	42.69, 42.69, 271.03	42.87, 42.87, 267.64
α, β, γ (°)	90, 90, 120	90, 90, 120	90, 90, 120
Resolution (Å)	2.35 (2.41–2.35)	2.66 (2.75–2.66)	2.80 (2.89–2.80)
*R*_merge_ a	5.6 (97.8)	12.7 (216.6)	14.3 (97.0)
*I*/σ*I*	22.0 (1.8)	14.5 (1.9)	7.7 (1.8)
Completeness (%)	99.8 (98.9)	100.0 (100.0)	100.0 (99.7)
Redundancy	7.6	17.4	4.9
**Refinement**			
Resolution (Å)	44.42–2.35	45.17–2.66	44.61–2.80
No. reflections	6362	4823	4172
*R*_work_ b/*R*_free_ c	18.9/25.5	21.1/24.0	21.1/27.1
No. atoms			
Protein	1005	1029	1032
Ligand	-	36	-
Water	38	24	45
*B*-factors (Å^2^)			
Protein	79	82	72
Ligand	-	97	-
Water	66	74	57
RMS deviations			
Bond lengths (Å)	0.010	0.009	0.008
Bond angles (°)	1.4	1.0	1.0

a R_merge_ = Σ*_h_*Σ*_j_* |I*_h,j_*−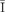 *_h_*|/Σ*_h_*Σ*_j_*|I*_h,j_*|, where I*_h,j_* is the *j*th observation of reflection *h*.

b R_work_ = Σ*_h_||*F*_oh_*|−|F*_ch_*||/Σ*_h_|*F*_oh_*|, where F*_oh_* and F*_ch_* are the observed and calculated structure factor amplitudes respectively for the reflection *h*.

c R_free_ is equivalent to R_work_ for a 5% subset of reflections not used in the refinement.

Numbers in parentheses refer to the outer resolution shell.

### Structure Determination

Data were processed using XDS [61] and Scala [62], [63], and the apo Keap1 BTB structure was solved by molecular replacement using Phaser [64] with the coordinates of the BTB domain from the SPOP–Cul3 complex [50] as a model (PDB accession code 4eoz). The structure was refined using Refmac [65] and manual model building was carried out using COOT [66]. The loop containing residues 114–118 showed a high degree of disorder, and model building in this region is tentative due to relatively weak and discontinuous electron density. The structures of the C151W mutant and the CDDO complex were solved using the structure of the apo BTB structure as a starting model, and subsequently refined using BUSTER [67]. Generation of the BUSTER restraint dictionary for the CDDO covalent adduct, and subsequent structure refinements were carried out within Astex's internal automated protein-ligand structure determination pipeline [68]. Refinement statistics are presented in Table 1. Coordinates and structure factors for structures presented here have been deposited with the Protein Data Bank [69] with accession codes 4cxi (apo), 4cxt (CDDO) and 4cxj (C151W).

### AlphaScreen proximity assay

30 nM Keap-BTB (residues 35–235) was incubated with CDDO for 2.5 h at 37°C in black 384 well assay plates, followed by 30 nM biotinylated Cul3/Rbx1 for 1.5 h at room temperature. 20 µg/mL AlphaLISA Nickel chelate donor beads were then added for 30 min, followed by 20 µg/mL Alphascreen streptavidin acceptor beads (PerkinElmer). The plate was then incubated overnight at room temperature before reading using the Envision plate reader (PerkinElmer). An identical procedure was used for both C151 and C151S constructs. The signal observed with varying concentrations of CDDO is reported relative to a DMSO control, and is the mean of three measurements.

## Supporting Information

File S1 **Supporting information and figures.** Details of thermal denaturation assay and Epic BTB-Cul3 direct-binding assay.
(DOCX)
