# Supplementary material for: Structure of the BTB Domain of Keap1 and Its Interaction with the Triterpenoid Antagonist CDDO
Source: PLoS One. 2014 Jun 4;9(6):e98896. doi: 10.1371/journal.pone.0098896 (PMC4045772; doi:10.1371/journal.pone.0098896)
Supplement: File S1 — Supporting information and figures. Details of thermal denaturation assay and Epic BTB-Cul3 direct-binding assay. (DOCX) [file pone.0098896.s001.docx]

**Supporting Information S1**

***Thermal denaturation assay***

**Materials and Methods**

Thermal denaturation (T_m_) experiments in the presence of SYPRO orange dye (Invitrogen) were performed on a Mx3005P quantitative PCR instrument (Stratagene). Proteins were purified by Ni-affinity chromatography as described above (but without removal of the His-tag), and then dialysed overnight against 25 mM Tris/HCl pH 8.0, 150 mM NaCl, 1 mM TCEP. Samples (typically at ~0.5 mM) were diluted into an assay buffer (25 mM HEPES/NaOH pH 7.5, 100 mM NaCl) to a final concentration of 5 μM and SYPRO dye was added according to the manufacturer’s instructions. The plate was heated over a temperature range of 25-80°C and the temperature was increased by 1°C per minute. The fluorescence intensity was measured every 0.5°C.

**Results**

Comparison of the thermal denaturation profiles of wild-type and S172A Keap1 BTB proteins reproducibly showed a >3^o^C increase in melting temperature for the S172A mutant (data obtained for three sets of wild-type/S172A protein preparations, representative curve shown in Figure S1)


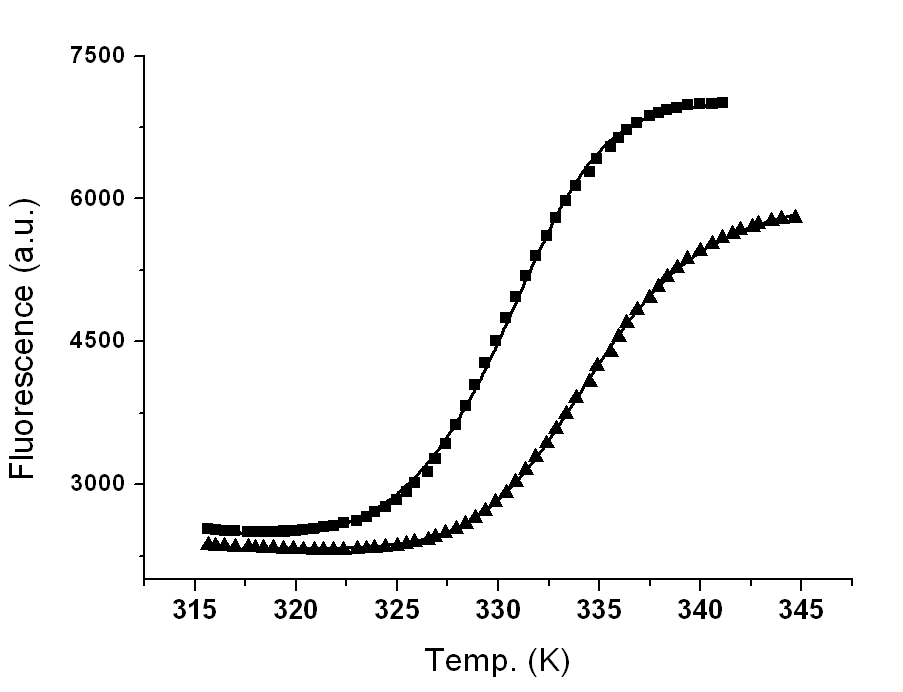


**Figure S1**  Representative thermal denaturation curves for wild-type (filled squares) and S172A mutant (filled triangles); a.u. = arbitrary units. Calculated T_m_ values are 329.8 K and 333.8 K respectively.

***Epic Keap1-Cul3 direct-binding assay***

**Materials and Methods**

His-Keap1 (residues 35-318) and His-Keap1 (residues 35-318) S172A proteins were overexpressed by baculovirus infection in Sf9 cells, and purified by affinity chromatography with Ni-NTA Superflow resin (Qiagen) and size exclusion chromatography on Superdex 75 (GE Healthcare). Protein-protein binding was measured as a function of the change in refractive index on a Corning Epic System. A streptavidin biosensor plate (Corning #5047) was pre-adapted with assay buffer (50 mM Tris, pH 7.5, 100 mM NaCl, 5 mM MgCl_2_, 0.5 mM TCEP, 0.005% bovine serum albumin, 2 mM CHAPS) for two hours at room temperature. Biotinylated Cul3/Rbx1 (10 µg/mL) was prepared as described in the main paper, and immobilized on the streptavidin in 20 µL of assay buffer for two hours at room temperature. The excess protein was removed with three washes of assay buffer, and allowed to equilibrate to room temperature with 5 µL of assay buffer per well. A baseline refractive index read was read on the Corning Epic System. Either Keap1 (35-318) or Keap1 (35-318) S172A were added to the wells at indicated concentrations in 15 µL of assay buffer and incubated for 30 min at room temperature. The refractive index was then read again on the Corning Epic System and the change in refractive index (measured as a shift in reflected wavelength) was plotted as a function of Keap1 concentration.

**Results**

Direct measurement of protein-protein binding indicated that the S172A point mutation imposes minimal change on the binding affinity of Cul3 for Keap1 (data obtained for three separate experiments, representative curves are shown in Figure S2). The observed K_d_ of the wild-type Keap1 construct for the Cul3 protein was 37 ± 4.9 nM, and 59.9 ± 5.2 nM for the corresponding S172A construct.

**Figure S2** Representative analysis of the label-free detection of Cul3 binding with the wild-type (open circles) and S172A mutant form (closed circles) of the Keap1 protein. Response is shown on the y-axis as a shift in reflected wavelength in picometers (pm).
